# Alcohol in Surgery

**Published:** 1902-11-29

**Authors:** 


### ALCOHOL IN SURGERY.

As in some respects complementary to, and in others contrasting strongly with, the opinions of Sir William Broadbent regarding the use of alcohol in medicine, an account of which we have given above, and also because of their own intrinsic importance, the views of Mr. Pearce Gould[1] as to the use of alcohol in surgery are of much interest. He speaks with great positiveness on the subject, regarding the administration of alcohol to surgical patients as productive not only of no good, but of definite harm in almost every kind of case ; indeed he practically limits the use of alcohol in surgery to its administration in cases of shock, and even then in only very small doses. For many years, he says, he has dispensed almost entirely with the use of alcohol as an aid in surgical treatment. As a student he saw it used, almost as a matter of routine, for every kind of surgical malady except head injuries, and in his early days he naturally followed the practice of his teachers. But as soon as he made trial for himself of the effect of withholding alcohol, he found how entirely overrated its value was. He regards the opinion which is so commonly held as to the special value of alcoholic stimulants in all forms of septic inflammation—erysipelas, pyæmia, septicæmia, and hectic fever—as being founded solely upon tradition, unsupported by any trustworthy evidence. By a comparison of cases in which alcohol has been given with those from which it has been withheld, he has come to the conclusion that there are no cases which require alcohol so little as the septic ones, and that there are few in which its influence is so wholly harmful. It dries the mouth, it furs the tongue, it clouds the intellect, it lessens the ability to take, digest, and assimilate food, and does nothing to lessen the tissue waste, to increase the elimination of poison, to maintain the strength of the heart, or to arrest the disease. Mr. Pearce Gould relates cases showing how successfully even the most extreme examples of septicæmia may be treated without alcohol, and quotes many authorities to show that in the lower animals the administration of alcohol increases their susceptibility to infection, adding that experience with patients suffering from various forms of infection coincides with the results obtained by experiment upon animals. "Not only are individuals who are addicted to alcoholic excess possessed of a much diminished resistance to all forms of infection, but the administration of alcohol to those already infected not only fails to add to their power to resist the disease, but even lessens that power." Of all the bad uses to which alcohol is put as a therapeutic agent none is worse, he says, than its employment in any form of infective disease, and even in cases of uncontrollable suppuration from large tuberculous abscesses, nothing but good is to be found from entirely withholding all alcohol, even though the discontinuance may be made abruptly in cases in which it has been taken for a long time and in large doses. It has been a question of much surgical interest whether the repair of wounds and convalescence from severe injuries are aided by the administration of stimulants, but both experimental evidence and a somewhat large experience go to show that the drug is of no value for such purposes.

Turning to the subject of cancer, as to which at the Middlesex Hospital they have an exceptional experience, Mr. Pearce Gould holds that in it also alcohol is injurious. It may increase the activity of the disease, and it often adds to the patient's pain. There are, however, some cases of advanced cancer in which alcohol may be used with good effect. It is sometimes found that a small quantity of well-diluted spirit, given at about the same time as the evening dose of morphine will secure better sleep than the morphine alone. Sometimes again, where there is entire distaste for food, a little beer or wine will help patients to take food, and so promote their comfort ; and in extreme cases, where recovery seems out of the question, it may be easier and kinder to continue a lifelong habit rather than break it, even though the habit be to some extent injurious.

In the treatment of shock and collapse, however, alcohol does seem to be of some service. Mr. Pearce Gould would not place it among the means of primary value in the treatment of these conditions. It certainly is not of the same value as the horizontal position, rest, external heat, morphine, and possibly strychnine, nor does it ever produce such a striking effect as the introduction of normal saline fluid into the blood ; still alcohol is of use in shock in helping to tide over an acute emergency. For this purpose it should be given in small quantity, if possible well diluted, and its administration should not be continued after any improvement in the pulse is noticed. "Its use should be limited to a single dose, though of course that single dose may be divided up and even administered through more than one channel."

[1] Practitioner, Nov.

